# Minimal detectable change for mobility and patient-reported tools in people with osteoarthritis awaiting arthroplasty

**DOI:** 10.1186/1471-2474-15-235

**Published:** 2014-07-11

**Authors:** Justine M Naylor, Andrew Hayen, Edward Davidson, Danella Hackett, Ian A Harris, Gihan Kamalasena, Rajat Mittal

**Affiliations:** 1 Orthopaedic Department, Liverpool Hospital, Sydney, Australia; 2 School of Public Health and Community Medicine, University of New South Wales, Sydney, Australia; 3 Ingham Institute of Applied Medical Research, Sydney, Australia; 4 Physiotherapy Department, Nepean Hospital, Sydney, Australia; 5 Physiotherapy Department, Fairfield Hospital, Sydney, Australia

**Keywords:** Osteoarthritis, Arthroplasty, Clinimetrics, Patient-reported outcome measures, Timed mobility tests

## Abstract

**Background:**

Thoughtful use of assessment tools to monitor disease requires an understanding of clinimetric properties. These properties are often under-reported and, thus, potentially overlooked in the clinic. This study aimed to determine the minimal detectable change (MDC) and coefficient of variation per cent (CV%) for tools commonly used to assess the symptomatic and functional severity of knee and hip osteoarthritis.

**Methods:**

We performed a test-retest study on 136 people awaiting knee or hip arthroplasty at one of two hospitals. The MDC_95_ (the range over which the difference [change] for 95% of patients is expected to lie) and the coefficient of variation per cent (CV%) for the visual analogue scale (VAS) for joint pain, the six-minute walk test (6MWT), the timed up-and-go (TUG) test, the Knee Injury and Osteoarthritis Outcome Score (KOOS) and the Hip Disability and Osteoarthritis Outcome Score (HOOS) subscales were calculated.

**Results:**

Knee cohort (n = 75) - The MDC_95_ and CV% values were as follows: VAS 2.8 cm, 15%; 6MWT 79 m, 8%; TUG +/-36.7%, 13%; KOOS pain 20.2, 19%; KOOS symptoms 24.1, 22%; KOOS activities of daily living 20.8, 17%; KOOS quality of life 26.6, 44. Hip cohort (n = 61) - The MDC_95_ and CV% values were as follows: VAS 3.3 cm, 17%; 6MWT 81.5 m, 9%; TUG +/-44.6%, 16%; HOOS pain 21.6, 22%; HOOS symptoms 22.7, 19%; HOOS activities of daily living 17.7, 17%; HOOS quality of life 24.4, 43%.

**Conclusions:**

Distinguishing real change from error is difficult in people with severe osteoarthritis. The 6MWT demonstrates the smallest measurement error amongst a range of tools commonly used to assess disease severity, thus, has the capacity to detect the smallest real change above measurement error in everyday clinical practice.

## Background

Though there is no gold standard for monitoring the progression of knee or hip osteoarthritis (OA), there is value in monitoring the disease [1,2]. Knowledge of the trajectory of disease progression provides clinicians and patients with benchmarks against which the effectiveness of everyday self-management strategies [3] or clinician-provided interventions can be evaluated [1,2]. Furthermore, the timing of knee or hip arthroplasty for people with OA may also be informed by capturing significant deterioration in various health domains of those waitlisted for surgery when wait times are protracted. For example, those waitlisted for surgery may be escalated if there is documented evidence of significant decline since first consenting to the procedure.

There are multiple tools available which capture disease severity based on the symptoms and impairments associated with OA. These include tests of physical function and mobility as well as patient-reported surveys. Thoughtful monitoring of the clinical severity of OA in an individual using these tools requires knowledge of what changes measured by these tools can be considered real. In order to arrive at a decision, the clinician must first be cognisant of the minimum change measured by a given tool that is considered to be more than simple measurement error [4]. This minimum change is referred to as the minimal detectable change (MDC) [5-7] or smallest detectable change (SDC) [4,8,9] and is mathematically (linearly) related to the error of the measurement. Put simply, the MDC or the SDC reflects the smallest within-person change in score that can be interpreted as real and statistically significant [8]. In terms of clinimetrics, the MDC is a metric for reproducibility (specifically, a measure of agreement), and is determined by performing repeat measurements on patients over a short period of time [8]. The short time interval renders significant clinical change between assessments unlikely [8], and it also avoids the problem of response shift - a change in the meaning or a recalibration of an outcome - if the tool captures a patient-reported outcome [10].

Despite their obvious value in interpreting real change at the level of the individual, several recent reviews of tools used to assess OA and arthroplasty patients suggest that the measurement error and MDC for the tools are under-reported or underexplored [11-16]. This study, therefore, aimed to evaluate the MDC of tools commonly used to assess the symptomatic and functional severity of knee or hip OA. Specifically, using a test-retest design, we determined the MDC for the visual analogue scale for pain (VAS Pain) [1,17,18], the timed up-and-go (TUG) test [19], the six-minute walk test (6MWT) [20], the Knee Injury and Osteoarthritis Outcome Score (KOOS) [21] and the Hip Disability and Osteoarthritis Outcome Score (HOOS) [22] in individuals with severe OA. As a secondary aim, we also compared the MDCs for the different tools as the magnitude of error may influence the choice of tool used. The tools included were chosen by a multidisciplinary working party overseeing a State-wide program primarily intended to screen, treat and monitor patients with severe knee or hip OA waitlisted for arthroplasty surgery - the Osteoarthritis Chronic Care Program (OACCP) [23].

## Methods

### Study design and participants

A test-retest study involving individuals waitlisted for total knee arthroplasty (TKA) or total hip arthroplasty (THA) at one of two teaching hospitals was undertaken. Individuals meeting the following eligibility criteria were invited to participate: OA in the index joint; waitlisted within the previous two months; and willingness to attend two assessments separated by a 1-week interval. Participants were ineligible if they were unable to comprehend the study protocol either because of an English language limitation or because of documented dementia, or if they intended to change pharmacological or physical management of their OA within the next week. Participants who reported an exacerbation of symptoms or had an acute illness at the first or second assessment were also subsequently deemed ineligible. The study was approved by The Nepean Blue Mountains Lead Human Research Ethics Committee and all participants provided written, informed consent.

### Study protocol

As per current practice, administrative staff contacted all waitlisted individuals via telephone to provide them with an appointment to the chronic care program for assessment of their joint status and overview of their arthritis management. Those meeting the inclusion criteria for the study were invited to be assessed twice for the purposes of the study. Those agreeing to participate were subsequently screened again by a researcher at the first assessment. Co-morbidities, medication lists, and radiology reports were reviewed as per the screening protocol for the OACCP [23]. Eligible participants then completed timed walk tests and several patient-reported measures. Simple yet standardised instructions regarding the completion of the walk tests and patient-reported measures were given as per the program’s procedure manual. The participant was instructed to interpret the surveys in the same way when they repeated them the following week and to wear the same footwear. As recommended for test-retest studies [4], the participant was scheduled another appointment one week later, with a maximum time between appointments of 10 days. A physiotherapist at each hospital attached to the waitlist assessment clinic undertook the second assessment, following the same testing procedures. By having a different tester undertake the second assessment, observer independence (from first to second measurement) was ensured [24,25]. Time of testing (morning or afternoon), as per usual clinical practice, was not standardised. Recruitment and testing of participants were staggered across the two sites such that both were completed at the first hospital prior to their commencement at the second.

### Specific tests

#### VAS for joint pain

Numerical scales for pain ranging from 0–10 appear to have fairly consistent interpretation across disease states [1]. Significant disability appears to emerge at scores greater than five (moderate degrees of pain) [1]. Participants were asked to mark the pain they felt in their index joint on average over the past week on a 10 cm scale anchored by ‘none’ to ‘extreme’. On both weeks, this was completed prior to the walk tests.

#### Timed mobility

Timed mobility was assessed using the TUG [19] and the 6MWT [20]. Both these tests are recognised performance-based tests for people with OA or who have undergone TKA or THA [14,16,26-28]. The TUG was assessed using an armchair (45 cm seat height); the time taken (seconds) to stand from sitting, walk three 3 meters as fast and safely as possible with or without a walking aid, turn, return to the chair and sit down was assessed. A minimum two tests was performed with the fastest time included in the analysis. The 6MWT was conducted on a 30 m flat track. Participants were instructed to perform each lap ‘as fast, but safely as possible’ and asked not to stop at each end unless a rest was required [29]. Participants walked alone unless they were deemed unsafe; walking aids were permitted if aids were typically used. The assessor provided standardised verbal encouragement at the end of each 2-lap set. As recommended [20], a practise 6MWT was conducted. This occurred prior to the completion of the surveys. A second test (the test to be included in the analysis as the Week 1 test) was conducted a minimum 30 minutes later, the definitive time dependent upon the individual’s symptomatic recovery. Distance covered was recorded in meters.

#### Joint-specific surveys

Patient-reported outcomes are commonly used to capture joint-specific pain and function. The KOOS [21] and the HOOS [22] were used here, both derived from the Western Ontario and McMaster Universities Osteoarthritis Index (WOMAC) and developed to capture higher level improvement in younger or more active knee or hip patients. Both surveys have been shown to have face and construct validity and are responsive across a range of conditions [12,13,21,22,30]. Whilst the aforementioned VAS for pain was used to capture average joint pain experienced across the week, the KOOS and HOOS capture pain (and impairment) in specific contexts. The surveys include 42 (KOOS) and 40 (HOOS) items covering five patient-relevant joint-related health dimensions referenced to ‘the last week’: Pain (KOOS 9 items, HOOS 10 items), Other Disease-Specific Symptoms (7,5), Activities of daily living (ADL) Function (17,17), Sport and Recreation Function (5,4), and joint-related Quality of Life (QOL) (4,4). Each item’s response is framed within a 5-point Likert scale, ranging from 0 (No Problems) to 4 (Extreme Problems). Each of the five scores is calculated as the sum of the items included. Within each dimension, scores are transformed to a 0–100 scale, with zero representing extreme joint problems and 100 representing no joint problems. In the present study, a priori, the Sport and Recreation Function dimension was excluded based on the knowledge (our own experience and the experience of the developers [21]) that few people wait-listed for arthroplasty engage in higher-level recreational activity, consequently the items (particularly for the KOOS) are generally viewed as less important compared to items in other dimensions [30].

Though not part of the OACCP, the Oxford Knee and Hip Scores (OKS, OHS) [31] were also added to the test protocol for research purposes. For brevity, the reproducibility results of the OKS and OHS are reported elsewhere [32].

#### Sample size and statistical analyses

A minimum 50 subjects is a general recommendation for reproducibility studies [33]. Thus, a minimum 50 people with knee OA and 50 with hip OA were planned. As the rate of knee surgery is greater than that for hip surgery at both sites, we anticipated a greater sample for the knee cohort in the timeframe available for recruitment. The presence of systematic bias across the two weeks was investigated using paired t-tests and the relationship between variability (error) and raw score was inspected using Bland and Altman plots [24,25,31]. The MDC for the walk tests and survey scores between the two test days (Week 1 and Week 2) were determined as described below.

For each measure, we calculated the standard error of measurement (SEM) and the 95% confidence interval [34,35]. We also estimated the 90% confidence interval for a score, which is calculated as 1.645 × SEM; this measure can be used to obtain a 90% confidence interval for an individual’s measurement. Then, for each measure, we calculated the MDC at two levels of confidence, MDC_90=_1.645 × √2× SEM, and MDC_95_ = 1.96 × √2× SEM .The MDC_90_ and the MDC_95_ indicate that the difference in two measurements for about 90% and 95% of patients respectively will lie in this range. We determined the values at two levels of confidence to aid comparison with the literature. We note that the MDC_95_ provides the same limits of agreement (LOA) as the Bland and Altman method for assessing agreement [8]. In order to compare agreement indices between tools, we calculated the co-efficient of variation per cent (CV%) using the SEM divided by the Week 1 average [36] multiplied by 100.

For all outcome variables we checked graphically that the distribution of measurements did not strongly violate the assumption of normality using histograms and Q-Q plots. We found a violation of this assumption for the variable QOL, which was strongly right-skewed. Due to the presence of zero values on the scale, we did not log-transform the QOL data to improve the distribution [25]. For TUG, using the Bland and Altman plots, we found that the variability in measurements was proportional to their level. Therefore, we log-transformed the TUG data [25] and we estimated the MDC_90_ and MDC_95_ based on this. In this case, the MDC_90_ and MDC_95_ provide an interval in which the *ratio* of the two measurements for 90% and 95% of patients will lie. We also calculated the coefficient of variation for TUG as $\sqrt{\exp\left(\mathrm{SE}M^{2}\right)-1}$, where the SEM was calculated using the log-transformed data. Finally, we re-estimated the MDC_90_ and MDC_95_ for TUG after omitting an outlying observation (for which TUG was 99.9 s for one hip cohort measurement).

All analyses were conducted using Stata version 13.1, College Station, TX and the study adhered to the guidelines for qualitative research (http://www.biomedcentral.com/authors/rats).

## Results

Over the study period (July – October 2011 Hospital 1, October 2011 – May 2012 Hospital 2), 260 people (n = 187 knee, n = 73 hip) were waitlisted for surgery. One hundred and ninety-five were eligible to participate; 148 of these consented (n = 80 knee, n = 68 hip) and 47 were unable due to work commitments or transport limitations. Sixty-five were ineligible (n = 17 non – OA, n = 48 non-English speaking). Of those who provided consent, 136 (n = 75 knee; n = 61 hip) attended both assessment sessions; 12 people did not have their second assessment due to illness or transport unavailability. The characteristics of the retained cohort and those for whom repeat data were not available were similar (Table 1). The demographic and health profile of the definitive cohort are summarised in Table 2.

**Table 1:** Retained vs Lost cohort

	**Retained cohort – two assessments N = 136**	**Lost cohort – one assessment N = 12**	P-value*
Age, yr, mean (sd)	66.5 (9.8)	67.8 (8.6)	0.62
Female, n (%)	80 (59)	9 (75)	0.36
Body Mass Index, kg/m^2^, mean (sd)	32.5 (7.0)	32.7 (8.8)	0.94
Timed Up-and-Go, s	14.0 (8.1)	16.7 (10.6)	0.61
Six-minute walk test, m	332.2 (112.5)	250.2 (84.9)	0.10
Visual analogue scale, Pain (index joint), cm	6.7 (2.0)	6.4 (1.6)	0.46

*Independent sample t-tests or Fisher’s Exact Test; sd, standard deviation.

**Table 2:** Demographic and health profile of the definitive cohort

	**Knee osteoarthritis N = 75**	**Hip osteoarthritis N = 61**
Age, years, mean (sd)	67.6 (9.4)	65.2 (10.2)
Female, n (%)	47 (63)	33 (54)
Body Mass Index, kg/m^2^, mean (sd)	33.7 (7.4)	30.9 (6.4)
At least 1 co-morbidity, n (%)	61 (83)	54 (89)
Cardiovascular	50	43
Diabetes Mellitus	20	14
Thyroid	8	4
Respiratory	6	9
Central nervous system	2	4
Kidney	2	2
Other	13	16
Other lower limb or lumbar spine impairment	39 (52%)	29 (48%)
Previous knee or hip arthroplasty	17 (23%)	12 (20%)

### Practise 6MWT and missing data

One hundred and fifteen participants (68 of 75 knee participants, 47 of 61 hip participants) (85%) performed a practise 6MWT on the first testing day prior to undertaking the ‘included’ Week 1 6MWT; 21 were unwilling to repeat the test on the same day. There were no significant differences between the practise and included 6MWTs for either the knee [346.8 (101.0) vs 350.9 m (104.0), p = 0.34] or hip cohort [347.5 m (109.7) vs 343.5 (108.0), p = 0.28]. Thus, participants who did not complete a practise test remained included in the week-to-week analyses.

Complete week-to-week data sets were not available for all 136 participants. Ten participants (n = 10) refused to repeat the 6MWT or the TUG the second week and some did not complete every survey or VAS pain scale at the second assessment due to an administration error. Of those who did complete all surveys, there were no occasions of missing data as all surveys were checked at the time. The minimum sample size analysed for any one tool was 68 and 54 for the knee and hip cohorts respectively.

### MDC

**Figure 1:**
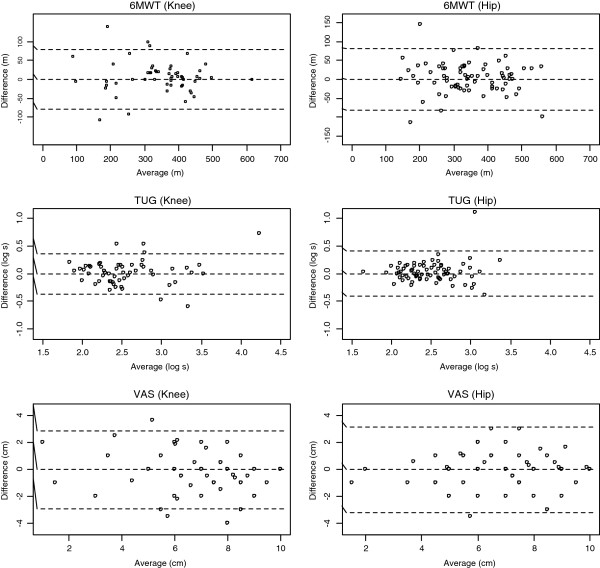
Bland and Altman Limits of Agreement for the six-minute walk (6MWT), timed up-and-go (TUG), and the visual analogue scale (VAS) for pain.

**Figure 2:**
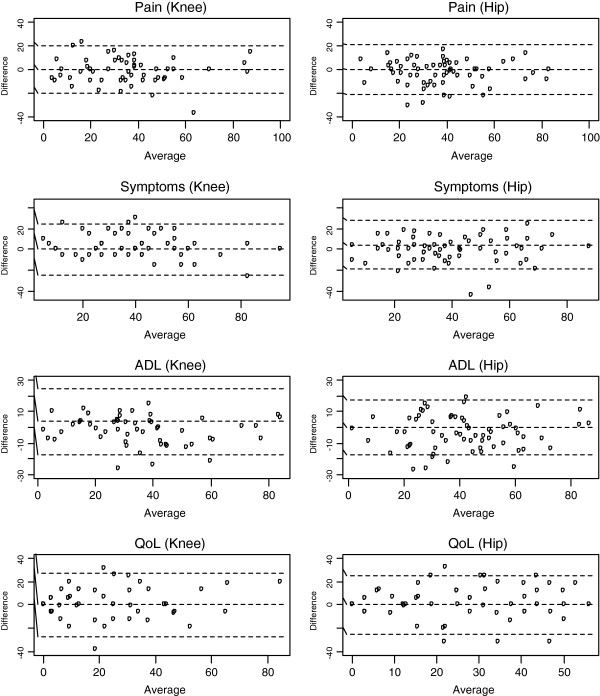
**Bland and Altman Limits of Agreement for the Knee Injury and Osteoarthritis Outcome Score (KOOS) and the Hip Disability and Osteoarthritis Outcome Score (HOOS) subscales.** Key: ADL – activities of daily living’ QoL – quality of life.

The error and agreement indices, including the SEM, the MDC and the CV% are summarised in Table 3. There were no or minor differences between the means of each test across the weeks (Table 3) and, with the exception of the TUG, the week-to-week differences were not related to the raw scores across the available range. Figures 1 and 2 illustrate the LOA for each tool assessed. The 6MWT demonstrated the lowest error from week-to-week for both the knee and hip cohorts; subsequently, the CV% (knee, 8%; hip, 9%) were the lowest. As the measurement error of TUG was related to level, we report the ratio (week 1 to week 2) in which 90% and 95% of measurements should lie. For knees, the MDC_90_ and MDC_95_ for TUG were ±30.8% and 36.7%, meaning that 90% and 95% of repeat measurements will be within about ±31% and 37% of the original measurement in stable patients respectively. For hips, the MDC_90_ and MDC_95_ were ±37.5% and 44.6%, meaning that 90% and 95% of repeat measurements for TUG will be within ±38% and 45% of the original measurement in stable patients, respectively. After removing the patient’s measurements who had a TUG of 99.9 seconds, the MDC_90_ and MDC_95_ was ±34.2% and 40.7%.

**Table 3:** Agreement and reliability indices of objective or patient-reported outcome measures

	**Week 1 mean (sd)**	**Week 2 mean (sd)**	**Difference mean (sd)**	**SEM (95% CI)**	**90% confidence in score**	**MDC**_**90**_	**MDC**_**95**_	**CV%**
Knee								
6MWT (m) n = 72	344.9 (105.6)	348.4 (102.3)	-3.5 (40.3)	28.5 (24.5 to 34.1)	46.9	66.3	79.0	8
TUG* (s), n = 74	12.7 (4.7)	13.1 (5.4)	-0.4 (3.7)	NA	NA	±30.8%	±36.7%	13
VAS Pain (cm), n = 71	6.8 (2.0)	6.8 (1.9)	0.1 (1.6)	1.0 (0.9 to 1.2)	1.7	2.4	2.8	15
KOOS, n = 68								
Pain	39.0 (18.7)	36.7 (18.2)	2.3 (10.3)	7.3 (6.2 to 8.8)	12.0	17.0	20.2	19
Symptoms	38.8 (19.2)	39.1 (18.8)	-0.4 (12.3)	8.7 (7.4 to 10.5)	40.0	20.2	24.1	22
ADL	43.8 (18.6)	40.2 (19.1)	3.6 (10.6)#	7.5 (6.4 to 9.0)	12.3	17.4	20.8	17
QOL	21.7 (7.7)	23.8 (18.8)	-2.7 (13.8)	9.6 (8.2 to 11.6)	15.9	22.4	26.6	44
Hip								
6MWT (m), n = 54	339.8 (107.8)	347.3 (105.3)	-7.5 (41.6)	29.4 (24.7 to 36.3)	57.7	68.5	81.5	9
TUG* ( s), n = 56	14.0 (7.2)	13.8 (6.6)	0.13 (3.6)	NA	NA	±37.5%	±44.6%	16
VAS Pain (cm), n = 58	7.1 (2.3)	6.8 (1.9)	0.4 (1.6)	1.2 (1.0 to 1.4)	1.9	2.7	3.3	17
HOOS, n = 56								
Pain	36.0 (20.9)	34.4 (20.6)	1.6 (11.0)	7.8 (6.6 to 9.6)	12.8	18.1	21.6	22
Symptoms	42.7 (19.4)	38.1 (21.8)	4.6 (11.6)#	8.2 (6.9 to 10.1)	13.5	19.2	22.7	19
ADL	36.7 (20.9)	34.9 (19.7)	1.8 (9.0)	6.4 (5.4 to 7.8)	10.5	14.8	17.7	17
QOL	20.4 (20.2)	21.8 (21.6)	-1.4 (12.5)	8.8 (7.5 to 10.9)	14.6	20.6	24.4	43

Key: SEM, standard error of measurement with 95% confidence level; MDC_90_ and MDC_95_, minimal detectable change at the 90% and 95% confidence level; CV%, co-efficient of variation per cent; 6MWT, six-minute walk test; TUG, timed up-and-go; VAS, visual analogue scale; KOOS, Knee Injury and Osteoarthritis Outcome Score; HOOS, Hip Disability and Osteoarthritis Outcome Score; ADL, activities of daily living; QOL, quality of life; NA, not applicable (The SEM for TUG is not provided as it is not derived using the same formula due to its skewed distribution); *TUG data were log-transformed - the corresponding MDC_90_ and MDC_95_ are then interpreted as 90% and 95% of the measurements being within a ratio of the original measurement. Note, average Week 1 6MWT above slightly differs from average Week 1 reported for the comparison between practise and official Week 1 as people who did not have a practise test are included in the average for Week 1 above; #, significantly different, p < 0.05.

## Discussion

Given the complexities and associated burden of quantifying change from one visit to the next [37], one could argue why not simply ask the patient if they have improved or worsened. Though the latter approach may be appealing, in cases where patients may benefit from reporting deterioration - for example, by being escalated to arthroplasty surgery - transparency and quantification of change are required. Further, protracted periods between assessments will undermine both patient and clinician recall, thus, one’s ability to recognise change will be in doubt [1]. Consequently, an objective method for interpreting ‘change’ is required.

The use of the MDC or other indices of error (or reproducibility) to determine thresholds for change is an objective, transparent, simple way to help the clinician monitor change in the individual with OA. Here, we provide the MDC for a range of tools with the 6MWT demonstrating (in a relative sense) the smallest measurement error across the physical and patient-reported tests assessed. For a change in these tools to be considered ‘real’, the patient with knee or hip OA respectively would need to demonstrate a minimum change (at the 95% level of confidence) of at least 79 and 81 m for the 6MWT, 3 cm for VAS Pain, 37% and 41% for TUG (from baseline), 20 and 22 for KOOS and HOOS Pain, 24 and 23 for KOOS and HOOS Symptoms, 21 and 18 for KOOS and HOOS ADL, and 27 and 24 for KOOS and HOOS QOL subscales.

The magnitude of the week-to-week variation and MDCs observed here are generally consistent with others. An earlier study observed that 90% of stable patients with musculoskeletal problems demonstrated a week-to-week change of up to 3-points (27%) in their pain rating on an 11-point numeric rating scale [38]. Kennedy et al. [28] reported the MDC_90_ for the TUG and 6MWT as 2.49 s and 61.34 m in their combined TKA and THA cohort. Our MDC_95_ for the KOOS subscales were generally smaller than the 95% LOA (equivalent to the MDC_95_) for those reported by Roos and Toksvig-Larsen [21] which ranged from 40 (Symptoms) to 60 (Sport and Recreation subscale), whilst our MDC_95_ for the HOOS subscales were slightly larger than those reported by Ornetti et al. [39] which ranged from 10 to 20. In terms of how the agreement indices of the KOOS and HOOS Pain, Symptom and ADL Function subscales compare with the OKS and OHS, we found that the CV% were similar (16% for both OKS and OHS) [32]. These observations are interesting as it appears the greater specificities afforded by the KOOS and HOOS subscales do not guarantee a smaller measurement error compared to a survey that does not differentiate contributions made by pain and functional impairment. It is noteworthy that Impellizzeri et al. [40] reported a much smaller CV (7%) for the OKS; this is in part explained by the reverse scoring method (12–60, with low scores denoting less pain and impairment) used in their study.

The MDCs are related to the size of the measurement error. Our study design does not allow us to determine whether the error we have observed is due to within-individual inconsistency in interpretation of survey questions or subclinical, random biological fluctuations in the case of timed walk or continuous pain scale measurements. Nevertheless, the fact remains that ‘noise’ in outcome measures – whether they be objectively or subjectively measured - will undermine the capacity to monitor disease progression, thus, knowledge of the MDC of each tool is important for reliable interpretation of disease status. Our study design also does not allow us to determine what changes are clinically relevant. Whilst the MDC_90_ and MDC_95_ values provide clinicians with a threshold about which ‘true’ change can be considered to have occurred with considerable confidence, these thresholds do not denote thresholds for clinically important changes. Reference to the minimal clinically important difference (MCID) or minimal important change is expected to assist the determination of whether change is clinically relevant [1,8]. This notwithstanding, it is unclear what these values are for all these tools and their interpretation is contentious given that the MCID appears to vary according to baseline severity and the scale used to determine it, and may be time-dependent [1]. For now, then, the MDCs provide a robust alternative for interpreting change in the clinic.

We acknowledge the strengths and limitations of our study. We examined a well-defined cohort likely to be representative of patients with severe OA waitlisted for arthroplasty. This contention is supported by the observations that: 1) the age (68 and 65 yrs), BMI (34 and 31), and gender (female, 63 and 54%) profiles of the knee and hip cohorts respectively, reflect those of the entire patient populations waitlisted for hip or knee arthroplasty at the two sites involved (age, 69 and 65 yrs; BMI, 34 and 30; female gender, 68 and 58%, knee and hip cohorts respectively) as per the data each site routinely collects for submission to the State’s arthroplasty registry (Arthoplasty Clinical Outcome Registry for NSW, ACORN), and; 2) the baseline physical and patient-reported characteristics of our cohorts reflect those reported elsewhere [30-32,39-41]. Our sample size exceeded the minimum recommended sample size for reproducibility studies, we therefore contend the error margins are credible estimates and not unduly influenced by an inadequate sample size. We tested reproducibility under usual care conditions, thus avoiding overly optimistic error estimates. We have provided reproducibility indices of a range of tools commonly used to assess knee and hip OA in the one study allowing comparisons across the tools. In terms of limitations, we relied on participant perception of their stability in their health status and we did not challenge their declarations that they did not change their medication or physical activity levels between the two test days. Our assumption around stability of health status was necessary as there is no known gold standard for assessing stability in OA [1]. We deliberately avoided the arbitrary use of stability in one of the tools, for example VAS joint pain, as the criterion for participant inclusion in the analysis as this assumes superior reproducibility of the chosen criterion over all others. Nevertheless, 90% (64/71) of the knee and 84% (48/61) of the hip cohort demonstrated a test-retest difference of ≤ 2 points in VAS pain (details not shown in Results). Importantly, these changes align with the weekly changes observed in the LEAP Trial in a cohort of patients with OA who were considered stable [42]. Further support for our contention that the participants were stable is found in the observations (results not shown) that a change in one measure was not reliably associated with a change in another, both in terms of magnitude or direction, suggesting that the changes were, by and large, ‘noise’. Regarding unchanged medication and activity levels, participants were aware that their waitlist assessment was to be conducted over two assessments and these would be used by the clinicians to inform their management whilst waiting for surgery. Thus, we contend participants were unlikely to have changed their management knowing that the intention of the assessment (at least from a clinical assessment perspective) was to assess the appropriateness of their current management and to provide a new plan if deemed necessary.

## Conclusion

Knowledge of the MDC values for physical performance and patient-reported tests commonly used to monitor the severity of OA is necessary for interpreting change within the individual in the context of daily clinical practice. The 6MWD demonstrated the smallest measurement error and, thus, has the capacity to detect the smallest real change above measurement error, making it (potentially) the preferred measurement tool.

## Abbreviations

6MWT: Six minute walk test; ADL: Activity of daily living (KOOS, HOOS subscale); CV%: Coefficient of variation per cent; HOOS: Hip disability and osteoarthritis outcome score; KOOS: Knee injury and osteoarthritis outcome score; LOA: Limits of agreement; MDC_90_: Minimal detectable change (90% confidence level - the difference in two measurements for about 90% of patients will lie in this range).; MDC_95_: Minimal detectable change (95% confidence level - the difference in two measurements for about 95% of patients will lie in this range); OA: osteoarthritis; OACCP: Osteoarthritis chronic care program; QOL: Quality of life (KOOS, HOOS subscale); SDC: Smallest detectable change; SEM: Standard error of measurement; TKA: Total knee arthroplasty; THA: Total hip arthroplasty; TUG: Timed up-and-go; VAS: Visual analogue scale

## Competing interests

The authors declare that they have no competing interests.

## Authors' contributions

JMN conceived and designed the study. JN, GK, RM, DH and ED contributed to data collection. AH and JN performed the analysis with input from IAH. JN and AH prepared the manuscript. All authors read, reviewed and approved the manuscript.

## Pre-publication history

The pre-publication history for this paper can be accessed here:

http://www.biomedcentral.com/1471-2474/15/235/prepub
